# Supplementary material for: OpenMM 7: Rapid development of high performance algorithms for molecular dynamics
Source: PLoS Comput Biol. 2017 Jul 26;13(7):e1005659. doi: 10.1371/journal.pcbi.1005659 (PMC5549999; doi:10.1371/journal.pcbi.1005659)
Supplement: S1 Source Code — (ZIP) [file pcbi.1005659.s002.zip › OpenMM-7.0.1-Source/docs/api-c++/generated/OpenMM.GBSAOBCForce.html]

GBSAOBCForce — OpenMM @OPENMM\_MAJOR\_VERSION@.@OPENMM\_MINOR\_VERSION@ documentation


# GBSAOBCForce¶

*class* `OpenMM::``GBSAOBCForce`¶
:   This class implements an implicit solvation force using the GBSA-OBC model.

    To use this class, create a `GBSAOBCForce` object, then call `addParticle()` once for each particle in the `System` to define its parameters. The number of particles for which you define GBSA parameters must be exactly equal to the number of particles in the `System`, or else an exception will be thrown when you try to create a `Context`. After a particle has been added, you can modify its force field parameters by calling `setParticleParameters()`. This will have no effect on Contexts that already exist unless you call `updateParametersInContext()`.

    When using this `Force`, the `System` should also include a `NonbondedForce`, and both objects must specify identical charges for all particles. Otherwise, the results will not be correct. Furthermore, if the nonbonded method is set to CutoffNonPeriodic or CutoffPeriodic, you should call setReactionFieldDielectric(1.0) on the `NonbondedForce` to turn off the reaction field approximation, which does not produce correct results when combined with GBSA.

    Methods

    |  |  |
    | --- | --- |
    | `GBSAOBCForce` | Create a `GBSAOBCForce`. |
    | `getNumParticles` | Get the number of particles in the system. |
    | `addParticle` | Add the GBSA parameters for a particle. |
    | `getParticleParameters` | Get the force field parameters for a particle. |
    | `setParticleParameters` | Set the force field parameters for a particle. |
    | `getSolventDielectric` | Get the dielectric constant for the solvent. |
    | `setSolventDielectric` | Set the dielectric constant for the solvent. |
    | `getSoluteDielectric` | Get the dielectric constant for the solute. |
    | `setSoluteDielectric` | Set the dielectric constant for the solute. |
    | `getSurfaceAreaEnergy` | Get the energy scale for the surface energy term, measured in kJ/mol/nm^2. |
    | `setSurfaceAreaEnergy` | Set the energy scale for the surface energy term, measured in kJ/mol/nm^2. |
    | `getNonbondedMethod` | Get the method used for handling long range nonbonded interactions. |
    | `setNonbondedMethod` | Set the method used for handling long range nonbonded interactions. |
    | `getCutoffDistance` | Get the cutoff distance (in nm) being used for nonbonded interactions. |
    | `setCutoffDistance` | Set the cutoff distance (in nm) being used for nonbonded interactions. |
    | `updateParametersInContext` | Update the particle parameters in a `Context` to match those stored in this `Force` object. |
    | `usesPeriodicBoundaryConditions` | Returns whether or not this force makes use of periodic boundary conditions. |

    Enum: NonbondedMethod

    |  |  |
    | --- | --- |
    | **NoCutoff** | No cutoff is applied to nonbonded interactions. The full set of N^2 interactions is computed exactly. This necessarily means that periodic boundary conditions cannot be used. This is the default. |
    | **CutoffNonPeriodic** | Interactions beyond the cutoff distance are ignored. |
    | **CutoffPeriodic** | Periodic boundary conditions are used, so that each particle interacts only with the nearest periodic copy of each other particle. Interactions beyond the cutoff distance are ignored. |

    `GBSAOBCForce`()¶
    :   Create a `GBSAOBCForce`.

    int `getNumParticles`() *const*¶
    :   Get the number of particles in the system.

    int `addParticle`(double *charge*, double *radius*, double *scalingFactor*)¶
    :   Add the GBSA parameters for a particle. This should be called once for each particle in the `System`. When it is called for the i’th time, it specifies the parameters for the i’th particle.

        |  |  |
        | --- | --- |
        | Parameters: |  |

        - **charge** – the charge of the particle, measured in units of the proton charge
        - **radius** – the GBSA radius of the particle, measured in nm
        - **scalingFactor** – the OBC scaling factor for the particle

        |  |  |
        | --- | --- |
        | Returns: | the index of the particle that was added |

    void `getParticleParameters`(int *index*, double &*charge*, double &*radius*, double &*scalingFactor*) *const*¶
    :   Get the force field parameters for a particle.

        |  |  |
        | --- | --- |
        | Parameters: |  |

        - **index** – the index of the particle for which to get parameters
        - **charge** – [out] the charge of the particle, measured in units of the proton charge
        - **radius** – [out] the GBSA radius of the particle, measured in nm
        - **scalingFactor** – [out] the OBC scaling factor for the particle

    void `setParticleParameters`(int *index*, double *charge*, double *radius*, double *scalingFactor*)¶
    :   Set the force field parameters for a particle.

        |  |  |
        | --- | --- |
        | Parameters: |  |

        - **index** – the index of the particle for which to set parameters
        - **charge** – the charge of the particle, measured in units of the proton charge
        - **radius** – the GBSA radius of the particle, measured in nm
        - **scalingFactor** – the OBC scaling factor for the particle

    double `getSolventDielectric`() *const*¶
    :   Get the dielectric constant for the solvent.

    void `setSolventDielectric`(double *dielectric*)¶
    :   Set the dielectric constant for the solvent.

    double `getSoluteDielectric`() *const*¶
    :   Get the dielectric constant for the solute.

    void `setSoluteDielectric`(double *dielectric*)¶
    :   Set the dielectric constant for the solute.

    double `getSurfaceAreaEnergy`() *const*¶
    :   Get the energy scale for the surface energy term, measured in kJ/mol/nm^2.

    void `setSurfaceAreaEnergy`(double *energy*)¶
    :   Set the energy scale for the surface energy term, measured in kJ/mol/nm^2.

    NonbondedMethod `getNonbondedMethod`() *const*¶
    :   Get the method used for handling long range nonbonded interactions.

    void `setNonbondedMethod`(NonbondedMethod *method*)¶
    :   Set the method used for handling long range nonbonded interactions.

    double `getCutoffDistance`() *const*¶
    :   Get the cutoff distance (in nm) being used for nonbonded interactions. If the NonbondedMethod in use is NoCutoff, this value will have no effect.

        |  |  |
        | --- | --- |
        | Returns: | the cutoff distance, measured in nm |

    void `setCutoffDistance`(double *distance*)¶
    :   Set the cutoff distance (in nm) being used for nonbonded interactions. If the NonbondedMethod in use is NoCutoff, this value will have no effect.

        |  |  |
        | --- | --- |
        | Parameters: |  |

        - **distance** – the cutoff distance, measured in nm

    void `updateParametersInContext`(Context &*context*)¶
    :   Update the particle parameters in a `Context` to match those stored in this `Force` object. This method provides an efficient method to update certain parameters in an existing `Context` without needing to reinitialize it. Simply call `setParticleParameters()` to modify this object’s parameters, then call `updateParametersInContext()` to copy them over to the `Context`.

        The only information this method updates is the values of per-particle parameters. All other aspects of the `Force` (the nonbonded method, the cutoff distance, etc.) are unaffected and can only be changed by reinitializing the `Context`. Furthermore, this method cannot be used to add new particles, only to change the parameters of existing ones.

    bool `usesPeriodicBoundaryConditions`() *const*¶
    :   Returns whether or not this force makes use of periodic boundary conditions.

        |  |  |
        | --- | --- |
        | Returns: | true if force uses PBC and false otherwise |

High performance molecular simulation on GPUs


### Navigation

- Core Objects
- Forces
- Integrators
- Extras

©2015, Stanford University and the Authors.
|
Powered by Sphinx 1.3.1
& Alabaster 0.7.7
|
Page source
